# The complete chloroplast genome of an annual halophyte herb, *Suaeda glauca* (Amaranthaceae)

**DOI:** 10.1080/23802359.2019.1659111

**Published:** 2019-09-06

**Authors:** Xiao-Jian Qu, Li-Kang Liu, Luo-Yan Zhang, Xue-Jie Zhang, Shou-Jin Fan

**Affiliations:** Key Lab of Plant Stress Research, College of Life Sciences, Shandong Normal University, Ji’nan, Shandong, China

**Keywords:** *Suaeda glauca*, plastome, phylogenomics

## Abstract

The complete chloroplast genome (plastome) of *Suaeda glauca*, an annual halophytic herb, was determined in this study. The plastome was 149,807 bp in size, containing a large single-copy region (82,162 bp), a small single-copy region (18,191 bp), and two inverted repeats regions (24,727 bp). The overall GC content of this plastome was 36.5%. In total, 113 unique genes, including 79 protein-coding genes (PCGs), 30 tRNAs and 4 rRNAs, were annotated. Phylogenomic analysis showed that *S. glauca* was sister to other *Suaeda* species.

*Suaeda glauca* (Amaranthaceae), one of the wild resources with incalculable ecological and economic benefit, is distributed in seashore salt marsh and inland of saline soil of China, Siberia, Korea and Japan (Duan et al. 2018). Like *S. salsa* (Chen et al. 2010; Song and Wang 2015), it is an annual halophytic herb with tolerance to salt. The genus *Suaeda* have been applied as model halophytes for understanding salt tolerance (Sui et al. 2010; Yang et al. 2010; Song et al. 2011; Li et al. 2012; Cheng et al. 2014; Guo et al. 2015; Wang et al. 2015; Chen et al. 2016; Song et al. 2016; Zhou et al. 2016; Song et al. 2017; Guo et al. 2018; Liu et al. 2018). Until to now, there are 19 of *ca*. 100 *Suaeda* species reported in China (Xing 2018). As one of the 19 *Suaeda* species in China, we reported the plastome of *S. glauca* for resolving its phylogenetic position.

Fresh leaves of *S. glauca* were collected from Hekou District (Shandong, China; 38°5'N, 118°40'E). Voucher specimen (hsdwz-1) was deposited at College of Life Sciences, Shandong Normal University. Total genomic DNA was extracted by the modified CTAB method described in Wang et al. (2013). Due to limited fresh sample, the plastid DNA was not directly extracted (Liu et al. 2017). The total genomic DNA was used for library preparation and paired-end (PE) sequencing by the Illumina MiSeq instrument at Novogene (Beijing, China). The plastome was assembled using Organelle Genome Assembler (OGA) described in Qu X-J (2019). Plastome annotation was conducted with Plastid Genome Annotator (PGA; Qu et al. 2019), coupled with manual correction using Geneious v9.1.4. To determine the phylogenetic placement of *S. glauca*, a maximum likelihood (ML) tree was reconstructed using RAxML v8.2.10 (Stamatakis 2014), including tree robustness assessment using 1,000 rapid bootstrap replicates with the GTRGAMMA substitution model, based on alignment of 79 shared PCGs using MAFFT v7.313 (Katoh and Standley 2013).

The complete plastome of *S. glauca* (GenBank accession number: MK867773) was 149,807 bp in size and contained a large single-copy region (LSC: 82,162 bp), a small single-copy region (SSC: 18,191 bp), and two inverted repeats regions (IR: 24,727 bp). The overall GC content was 36.5%. In total, 113 unique genes, including 79 protein-coding genes (PCGs), 30 tRNAs and 4 rRNAs were annotated. Among them, eleven PCGs and six tRNAs contained introns, in which nine PCGs and six tRNAs contained one intron and two PCGs contained two introns. There were 18 duplicated genes in the IR. The ML phylogenetic tree showed that *S. glauca* was sister to other *Suaeda* species (Figure 1).

**Figure 1.**
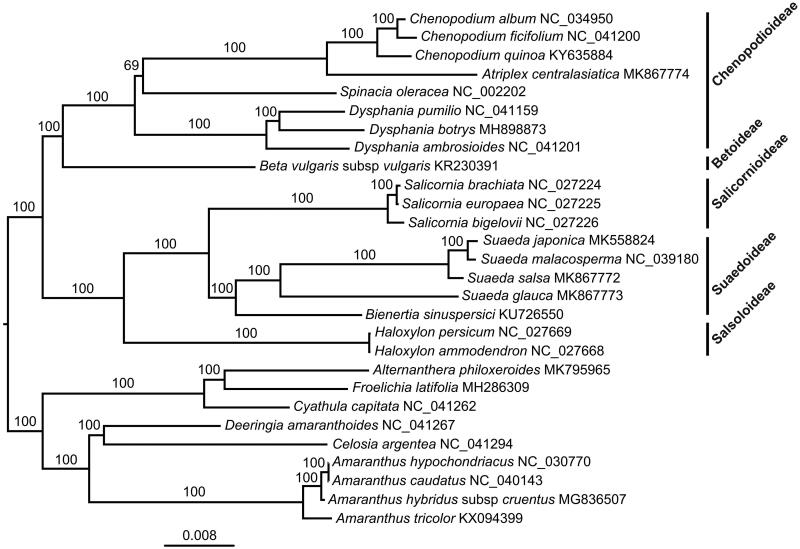
A maximum likelihood (ML) tree inferred from 79 plastome genes is shown. Four *Amaranthus* species, one *Celosia*, one *Deeringia*, one *Cyathula*, one *Froelichia*, and one *Alternanthera* from Amaranthaceae are used as outgroup. The numbers on branches are bootstrap support values.

## References

[CIT0001] Chen M, Song J, Wang BS 2010 NaCl increases the activity of the plasma membrane H^+^-ATPase in C3 halophyte *Suaeda salsa* callus. Acta Physiol Plant. 32(1):27–36.

[CIT0002] Chen TS, Yuan F, Song J, Wang BS 2016 Nitric oxide participates in waterlogging tolerance through enhanced adventitious root formation in the euhalophyte *Suaeda salsa*. Functional Plant Biol. 43(3):244–253. 10.1071/FP15120 32480457

[CIT0003] Cheng S, Yang Z, Wang MJ, Song J, Sui N, Fan H 2014 Salinity improves chilling resistance in *Suaeda salsa*. Acta Physiol Plant. 36(7):1823–1830.

[CIT0004] Duan HM, Ma YC, Liu RR, Li Q, Yang Y, Song J 2018 Effect of combined waterlogging and salinity stresses on euhalophyte *Suaeda glauca*. Plant Physiol Biochem. 127:231–237. 29621719 10.1016/j.plaphy.2018.03.030

[CIT0005] Guo JR, Li YD, Han GL, Song J, Wang BS 2018 NaCl markedly improved the reproductive capacity of the euhalophyte *Suaeda salsa*. Functional Plant Biol. 45(3):350–361. 10.1071/FP17181 32290958

[CIT0006] Guo JR, Suo SS, Wang BS 2015 Sodium chloride improves seed vigour of the euhalophyte *Suaeda salsa*. Seed Sci Res. 25(3):335–344.

[CIT0007] Katoh K, Standley DM 2013 MAFFT multiple sequence alignment software version 7: improvements in performance and usability. Mol Biol Evol. 30(4):772–780. 23329690 10.1093/molbev/mst010 PMC3603318

[CIT0008] Li X, Liu Y, Chen M, Song YP, Song J, Wang BS, Feng G 2012 Relationships between ion and chlorophyll accumulation in seeds and adaptation to saline environments in *Suaeda salsa* populations. Plant Biosystems. 146(sup1):142–149.

[CIT0009] Liu F, Jin Z, Wang Y, Bi YP, Melton JT 2017 Plastid genome of *Dictyopteris divaricata* (Dictyotales, Phaeophyceae): understanding the evolution of plastid genomes in brown algae. Mar Biotechnol. 19(6):627–637. 10.1007/s10126-017-9781-5 29164355

[CIT0010] Liu QQ, Liu RR, Ma YC, Song J 2018 Physiological and molecular evidence for Na^+^ and Cl^-^ exclusion in the roots of two *Suaeda salsa* populations. Aquat Bot. 146:1–7.

[CIT0011] Qu X-J 2019 Complete plastome sequence of an endangered species, *Calocedrus rupestris* (Cupressaceae). Mitochondrial DNA B. 4(1):762–763.

[CIT0012] Qu XJ, Moore MJ, Li DZ, Yi TS 2019 PGA: a software package for rapid, accurate, and flexible batch annotation of plastomes. Plant Methods. 15(1):50. 31139240 10.1186/s13007-019-0435-7 PMC6528300

[CIT0013] Song J, Shi G, Gao B, Fan H, Wang B 2011 Waterlogging and salinity effects on two *Suaeda salsa* populations. Physiol Plant. 141(4):343–351. 21214881 10.1111/j.1399-3054.2011.01445.x

[CIT0014] Song J, Shi WW, Liu RR, Xu YG, Sui N, Zhou JC, Feng G 2017 The role of the seed coat in adaptation of dimorphic seeds of the euhalophyte *Suaeda salsa* to salinity. Plant Species Biol. 32(2):107–114.

[CIT0015] Song J, Wang B 2015 Using euhalophytes to understand salt tolerance and to develop saline agriculture: *Suaeda salsa* as a promising model. Ann Bot. 115(3):541–553. 25288631 10.1093/aob/mcu194 PMC4332605

[CIT0016] Song J, Zhou JC, Zhao WW, Xu HL, Wang FX, Xu YG, Wang L, Tian CY 2016 Effects of salinity and nitrate on production and germination of dimorphic seeds applied both through the mother plant and exogenously during germination in *Suaeda salsa*. Plant Species Biol. 31(1):19–28.

[CIT0017] Stamatakis A 2014 RAxML version 8: a tool for phylogenetic analysis and post-analysis of large phylogenies. Bioinformatics. 30(9):1312–1313. 24451623 10.1093/bioinformatics/btu033 PMC3998144

[CIT0018] Sui N, Li M, Li K, Song J, Wang BS 2010 Increase in unsaturated fatty acids in membrane lipids of *Suaeda salsa* L. enhances protection of photosystem II under high salinity. Photosynthetica. 48(4):623–629.

[CIT0019] Wang HY, Jiang DF, Huang YH, Wang PM, Li T 2013 Study on the phylogeny of *Nephroma helveticum* and allied species. Mycotaxon. 125(1):263–275.

[CIT0020] Wang F, Xu YG, Wang S, Shi W, Liu R, Feng G, Song J 2015 Salinity affects production and salt tolerance of dimorphic seeds of *Suaeda salsa*. Plant Physiol Biochem. 95:41–48. 26184090 10.1016/j.plaphy.2015.07.005

[CIT0021] Xing JW 2018 Revision of the *Suaeda* in CHINA. Oceanol Limnol Sinica. 49(06):1375–1379.

[CIT0022] Yang MF, Song J, Wang BS 2010 Organ-specific responses of vacuolar H-ATPase in the shoots and roots of C halophyte *Suaeda salsa* to NaCl. J Integr Plant Biol. 52(3):308–314. 20377691 10.1111/j.1744-7909.2010.00895.x

[CIT0023] Zhou JC, Fu TT, Sui N, Guo JR, Feng G, Fan JL, Song J 2016 The role of salinity in seed maturation of the euhalophyte *Suaeda salsa*. Plant Biosystems. 150(1):83–90.

